# Porcine Endogenous Retrovirus (PERV) – Molecular Structure and Replication Strategy in the Context of Retroviral Infection Risk of Human Cells

**DOI:** 10.3389/fmicb.2018.00730

**Published:** 2018-04-11

**Authors:** Krzysztof Łopata, Emilia Wojdas, Roman Nowak, Paweł Łopata, Urszula Mazurek

**Affiliations:** ^1^Department of Molecular Biology, School of Pharmacy with the Division of Laboratory Medicine in Sosnowiec, Medical University of Silesia, Katowice, Poland; ^2^Department of Instrumental Analysis, School of Pharmacy with the Division of Laboratory Medicine in Sosnowiec, Medical University of Silesia, Katowice, Poland

**Keywords:** porcine endogenous retrovirus, xenotransplantation, PERV molecular structure, PERV biological cycle, PERV, PERV transmission risk

## Abstract

The xenotransplantation of porcine tissues may help overcome the shortage of human organs for transplantation. However, there are some concerns about recipient safety because the risk of porcine endogenous retrovirus (PERV) transmission to human cells remains unknown. Although, to date, no PERV infections have been noted *in vivo*, the possibility of such infections has been confirmed *in vitro*. Better understanding of the structure and replication cycle of PERVs is a prerequisite for determining the risk of infection and planning PERV-detection strategies. This review presents the current state of knowledge about the structure and replication cycle of PERVs in the context of retroviral infection risk.

## Introduction

Xenotransplantation, type of medical procedure that could potentially overcome the shortage of human organs for transplantation, relies on the use of domestic pigs as donors of organs and tissues. Xenotransplants could also serve as a temporary solution until an appropriate human donor is found. The possibility of individual transplant selection with regard to size and age represents an additional argument for the use of pigs as donors. Genetically modified pigs are a potential source of cells and tissues for the treatment of Parkinson’s disease, diabetes mellitus, and corneal opacity (Denner, 2016b; Cooper et al., 2017; Walters and Burlak, 2017). Currently, intensive research is being carried out on the utility of porcine kidney, heart, lung, and liver tissues for xenotransplantation (Cooper et al., 2017; Meier et al., 2017; Walters and Burlak, 2017). Porcine liver tissue could also serve as a source of modified hepatocytes for the treatment of patients with congenital metabolic pathologies in order to rebalance the level of hepatic enzymes. In addition, porcine hepatocytes could be used *ex vivo* in liver perfusion-assist devices (Cooper et al., 2017). Diabetes mellitus type 1 affects millions of people worldwide, resulting in immense treatment costs. Intensive research is currently focused on the transplantation of encapsulated islets of genetically modified pigs into humans (Cooper et al., 2017; Dhanasekaran et al., 2017). The application of genetically engineered porcine tissues can also serve as a temporary skin xenograft in the treatment of severe wounds (Nessler and Chrapusta, 2013; Scobie et al., 2013). In ophthalmology, clinical trials utilizing decellularized porcine corneas recellurized by autologous keratocytes are underway (Hara and Cooper, 2011; Kim and Hara, 2015). The pigs could also be considered as donors of erythrocytes in transfusiology (Cooper et al., 2017).

The immunological barriers responsible for transplant rejection, as well as those connected with the risk of the cross-species transmission of PERVs, are being systematically reduced. The introduction of specific mutations involves the elimination of antigens that cause hyper acute rejection (*α1,3-galactosylotransferase* gene knock-out animals) or introduction of genes regulating the functions of the complement system, such as *CD46, CD55*, and *CD59* (Ekser and Cooper, 2010; Luo et al., 2012; Reardon, 2015; Yang et al., 2015; Hryhorowicz et al., 2017). Today, there are about 25 known genetic modifications of the porcine genome, with some pigs expressing multiple manipulations (Zeyland et al., 2014; Cooper et al., 2017). Until recently, genetic modifications relied on the use of synthetic zinc finger nucleases (ZnF) and transcription activator-like effector nucleases (TALEN) (Meier et al., 2017). A recent milestone in the field involves the implementation of novel technologies using clustered regularly interspaced short palindromic repeats/CRISPR-associated protein 9 (CRISPR-Cas9) endonucleases targeting multiple genes in a single reaction (Yang et al., 2015; Niu et al., 2017). However, genetic engineering of pigs raises new questions. One important concern is the final effect of the multiple editions of porcine genes. How such editing will influence the donor and recipient organisms is yet to be determined.

According to the classification of the International Committee on the Taxonomy of Viruses (ICTV), PERVs belong to the Retroviridae family, Orthoretrovirinae subfamily, Gammaretrovirus genus, and Porcine type-C oncovirus species. They were first described in 1970 as virus-like particles resembling those seen in the baby hamster kidney (BHK-21) cell line and murine cells infected with murine leukemia virus (MLV) (Breese, 1970). PERVs are closely related to MLV, feline leukemia virus (FeLV), gibbon ape leukemia virus (GaLV), and koala retrovirus (KoRV) (Denner, 2008). The estimated age of PERVs is about 7.4–8.3 million years (Tonjes and Niebert, 2003; Niebert and Tonjes, 2005; Tang et al., 2016). Retroviruses are a virus family with single-stranded RNA (ssRNA) genomes characterized by the presence of reverse transcriptase (RT). This enzyme plays a central role in the replication cycle of retroviruses because it transcribes genomic RNA into double-stranded DNA (dsDNA), called provirus, which is subsequently integrated into the genome of the host cells. Based on the complexity of their genomes, retroviruses can be classified into two groups: those with simple genomes (alpharetroviruses, betaretroviruses, gammaretroviruses, and epsilonretroviruses) and those with complex genomes (lentiviruses, deltaretroviruses, and spumaviruses) (Weiss, 2006). The cells of somatic tissues are the primary targets of retroviral infection by the exogenous retroviruses circulating nowadays. In ancient times, retroviral infections affected the germ line cells, and proviral sequences had the possibility to be passed from one generation to another, becoming endogenous retroviruses (ERVs) (Weiss, 2006; Hayward, 2017). PERVs constitute an integral part of the porcine genome and are present in various proportions depending on pig breed, tissue type, and retrovirus subtype (Sypniewski et al., 2005; Yu et al., 2007; Ma et al., 2010b; Zhang et al., 2010a; Liu et al., 2011; Mazurek et al., 2013; Denner, 2016b). There are three replication-competent subtypes of PERVs: PERV-A, -B, and -C. PERV-A and -B are polytropic, capable of infecting both porcine and human cells (Denner, 2008). PERV-C is ecotropic, infecting only porcine cells (Takeuchi et al., 1998). However, PERV-A/-C, the result of the recombination of subtypes A and C, is more infectious to human cells than non-recombinant PERV-A (Harrison et al., 2004). The possibility of infecting human cells (so far only *in vitro*) raises concerns, especially in the context of the eventual use of porcine cells, tissues, and organs in xenotransplantation. Precise knowledge of PERVs’ molecular structure and replication cycle is thus necessary for the determination of infection risk and the creation of strategies for PERV detection (Denner, 2011; Argaw and Wilson, 2012; Gola and Mazurek, 2014; Kimsa et al., 2014a; Godehardt et al., 2015). This review presents the current knowledge about the structure and replication cycle of PERVs in the context of the retroviral infection risk of human cells.

## PERV Molecular Structure

### Genomic RNA

**FIGURE 1:**
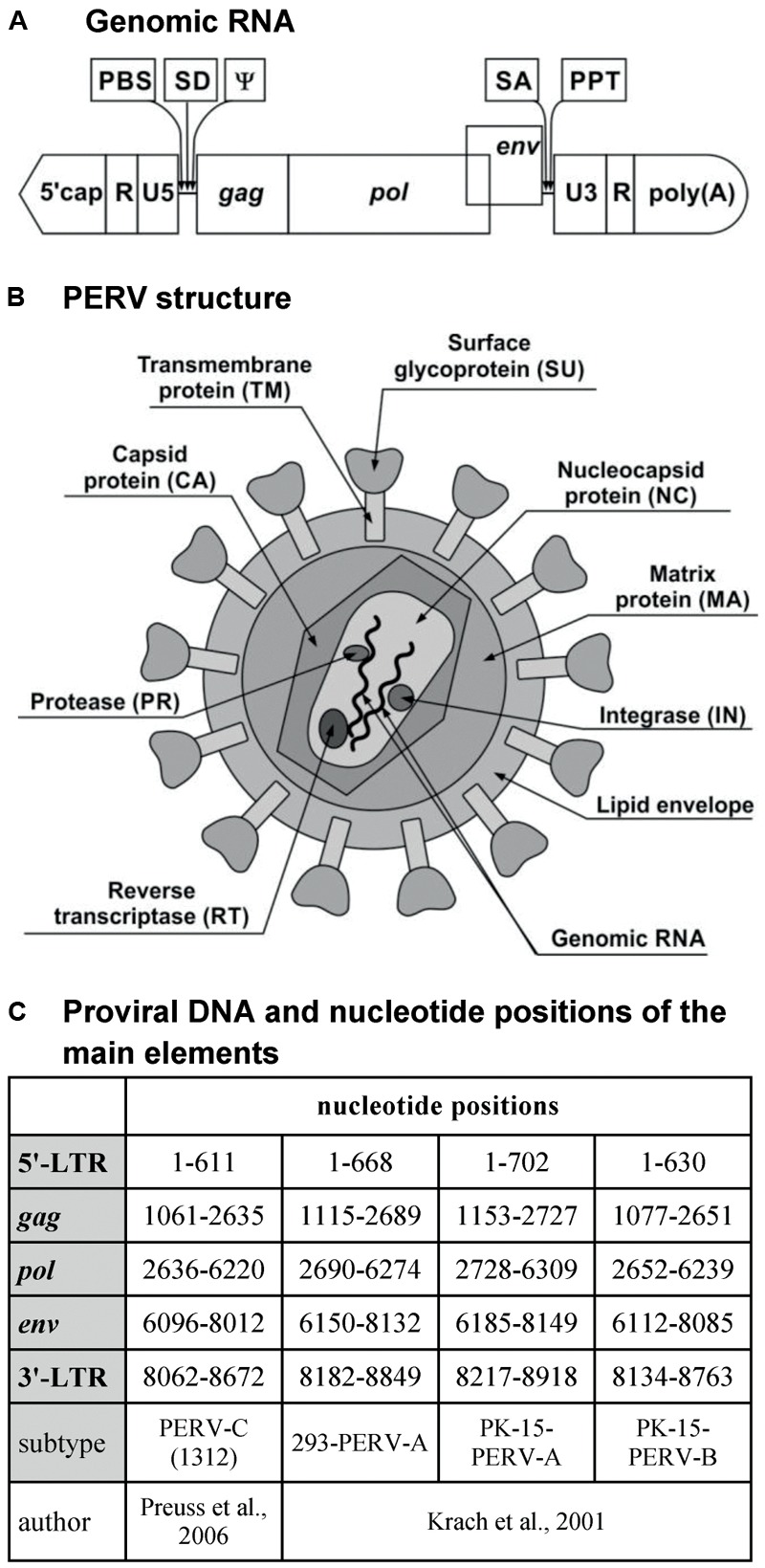
PERV. **(A)** Genomic RNA. **(B)** PERV structure. **(C)** Proviral DNA and nucleotide positions of the main elements. PBS, primer-binding site; SD, splice donor site; ψ, packaging signal psi; SA, splice acceptor site; PPT, polypurine tract; MA, matrix; CA, capsid; NC, nucleocapsid; PR, protease; RT, reverse transcriptase; IN, integrase; SU, surface envelope protein; TM, transmembrane envelope protein; LTR, long terminal repeat; *gag*, group-specific antigen; *pol*, polymerase; *env*, envelope.

The genomic RNA of PERV is composed of two identical single strands with positive polarity and includes both coding and non-coding sequences. The non-coding sequences are localized at both ends of the RNA, which includes the R and U5 regions at the 5′-end and the U3 and R regions at the 3′-end. Between non-coding sequences, there are sequences encoding the Gag, Pol, and Env proteins, that is, the *gag* (group-specific antigen), *pol* (polymerase gene), and *env* (envelope gene) genes, respectively (**Figure 1A**).

The *gag* gene encodes the structural proteins of the matrix (MA), the capsid (CA), and the nucleocapsid (NC) (**Figure 1B**). MA is associated with the inner lipid bilayer that descends from a host cell during budding. CA is the main structural protein of PERV, with a molecular weight of about 27 kDa. NC is the third structural protein, with a molecular weight of about 10 kDa (p10), and it is responsible for the efficient packaging of RNA in the virion (Akiyoshi et al., 1998; Dekker et al., 2003; Denner and Tonjes, 2012). In the case of gammaretroviruses, there is one additional protein localized in the Gag polyprotein between MA and CA. This is the p12 protein, which participates in the integration of the dsDNA within the genome of the host cell as well as in the release of new virus particles (Marcucci et al., 2008; Rein, 2011).

The *pol* gene encodes the following enzymes: protease (PR), RT, and integrase (IN). PR is a protein with a molecular weight of 14 kDa (p14) that catalyzes the proteolysis of the Gag and Pol polyproteins into the proteins described above (Czauderna et al., 2000; Blusch et al., 2002). RT is responsible for the transcription of viral ssRNA into dsDNA, which is subsequently incorporated into the genome of the host with the help of IN (Denner and Tonjes, 2012). In contrast to the Pol polyprotein, the products of the *gag* gene can be synthesized alone or as a single large polyprotein together with the products of the *pol* gene. The *gag* stop codon UAG is a part of the PR-coding sequence and can be read through by suppressor tRNA-accepting glutamine (Akiyoshi et al., 1998; Blusch et al., 2002).

The *env* gene encodes the proteins of the Env (Czauderna et al., 2000; Karlas et al., 2010). The Env protein is produced only from spliced *env* mRNA (Denner and Tonjes, 2012). It is synthesized as a single polyprotein, which is subsequently cleaved by a cellular furin-like PR into two components: the surface envelope protein SU (gp70) and the transmembrane envelope protein TM (p15E) (Akiyoshi et al., 1998; Lee et al., 2006; Chiang et al., 2007; Denner and Tonjes, 2012). Env glycoprotein has several glycosylation sites: about 10 in PERV-A, 6 in PERV-B, and 8 in PERV-C. Glycosylation may influence the binding to the host receptor (Lee et al., 2006, 2008b). The tropism of the retrovirus depends on the Env proteins. The SU protein is responsible for binding with the host receptor. The receptor-binding domain (RBD) is localized on the N-terminus of the SU protein and contains variable region A (VRA) and variable region B (VRB), localized between amino acids 96 and 126 and between amino acids 163 and 198 of the SU, respectively. The third region that is required for cellular binding is the proline-rich region (PRR), which is localized between amino acids 254 and 298 of the SU protein (Watanabe et al., 2005; Gemeniano et al., 2006; Denner, 2008). The last 100 amino acids of the SU protein are crucial to the binding and infectivity of PERV-C. Moreover, this region differs by only nine residues from the analogous region of PERV-A (Gemeniano et al., 2006). It has been demonstrated using the PERV-A/C envelope model that two single amino-acid substitutions can restore chimeric PERV-A/C’s ability to infect human cells to a titer equivalent to that of PERV-A. In addition, the tropism of vectors carrying PERV-C envelope mutants with only four amino acid changes in the C-terminus is similar to that of PERV-A (Argaw et al., 2008). The TM protein is buried in the lipid bilayer and anchors the SU protein to the surface of viral particles. The TM protein mediates the membrane fusion reaction (Bobkova et al., 2002; Chiang et al., 2007; Kubo et al., 2012). During the maturation of the virus, the TM protein is cleaved by PR to the p12E protein and R peptide. R peptide cleavage renders the virus capable of fusing with the cells of the host (Bobkova et al., 2002).

The primer-binding site (PBS), the sequence responsible for starting the first RNA strand-reverse transcription (RT), is located between the U5 region and *gag*. In the case of PERV-A and PERV-B, this sequence is complementary with glycine-tRNAs, and for PERV-C, it is complementary with proline-tRNAs. The splice donor (SD) site is situated downstream from the PBS sequence, followed by the packaging signal ψ (psi) (Choi et al., 2015). The splice acceptor (SA) site is located between the *pol* and *env* genes. The polypurine tract (PPT) is located between the *env* region and U3. PPT is required for RT as the primer for synthesis of the second strand of the DNA copy (Magre et al., 2003; Rein, 2011). The cap is situated on the 5′-side of the genomic RNA, while the 3′-end contains a polyA tail (Akiyoshi et al., 1998; Czauderna et al., 2000; Magre et al., 2003; Wilson et al., 2003; Rein, 2011).

### Provirus DNA

Porcine endogenous retrovirus, as an ERV, occurs mainly in the form of provirus integrated within the DNA of the host (pig). The length of the provirus is about 9000 bp (Czauderna et al., 2000; Krach et al., 2001; Preuss et al., 2006; Ma et al., 2010a; Tang et al., 2016). Just like the virus genome, the provirus contains coding sequences *gag, pol*, and *env*. These sequences are flanked by non-coding sequences, called long terminal repeats (LTRs), with U3, R, and U5 regions at both the 5′- and 3′-ends. The length of these LTRs is about 600–800 bp (**Figure 1C**; Wilson et al., 2003; Ma et al., 2010a). LTRs play an important role in the integration of the provirus within the host genome and the replication cycle of the virus. Moreover, they contain promoter, enhancer, and other regulator sequences important for the subsequent proviral transcription.

U3 appears to be the most heterogeneous region, with many binding sites for numerous transcription factors (Scheef et al., 2002; Denner et al., 2003; Wilson et al., 2003; Ha et al., 2007; Park et al., 2010; Jung et al., 2013b). In this region, there are direct repeated nucleotide sequences. Due to these repeats, we distinguish two types of LTRs. The first type is characteristic of PERV-A and -B and contains repeated 39 bp sequences. Each repeat consists of one 18 bp and one 21 bp subrepeat sequence (Krach et al., 2001; Scheef et al., 2001; Denner et al., 2003; Tonjes and Niebert, 2003; Zhang et al., 2010b). Various types of subrepeat sequences have been observed (Huh et al., 2007). The second type of LTR is characteristic of PERV-C and is composed of 37 bp repeats containing 18 bp fragments that are nearly identical to those found in PERV-A. The 37 and 39 bp repeats contain transcription factor-binding sites (Scheef et al., 2001; Denner et al., 2003; Wilson et al., 2003). A multimerization of these repeats in LTR correlates with an increase in virus titer. In the case of PERV-A/C (containing LTR from PERV-C), the number of 37 bp repeats increases up to the fifth passage on human 293 cells (Denner et al., 2003; Karlas et al., 2010). Three is the optimal number of 39 bp repeats for PERV-A and -B replication (Lee et al., 2012). The number of repeats is restricted and balanced by natural instability and the constraints imposed by virion packaging limits (Huh et al., 2007). In the replication of such a virus, a lower number of LTR repeats may reflect adaptation to the endogenous replication cycle, with a lower number of transcription factor-binding sites and lower transcriptional activity preventing damage to the host cells (Tonjes and Niebert, 2003). The increase in the number of repeats translates into an increase in the transcriptional activity of the retrovirus as well as an increase of the transcriptional activity of the neighboring host genes after the integration of the provirus (Denner et al., 2003). The transcriptional activity of PERVs may also be controlled by DNA methylation and by the inhibition of histone acetylation (Jung et al., 2008, 2013b; Park et al., 2010; Ha et al., 2012; Matousková et al., 2013; Wolf et al., 2013).

The R and U5 regions are conserved sequences with regulatory elements that can affect PERV transcription (Scheef et al., 2001; Wilson et al., 2003; Jung et al., 2013a). The deletion of the R region causes a significant increase in promoter activity (Scheef et al., 2002).

## PERV Replication Cycle

**FIGURE 2:**
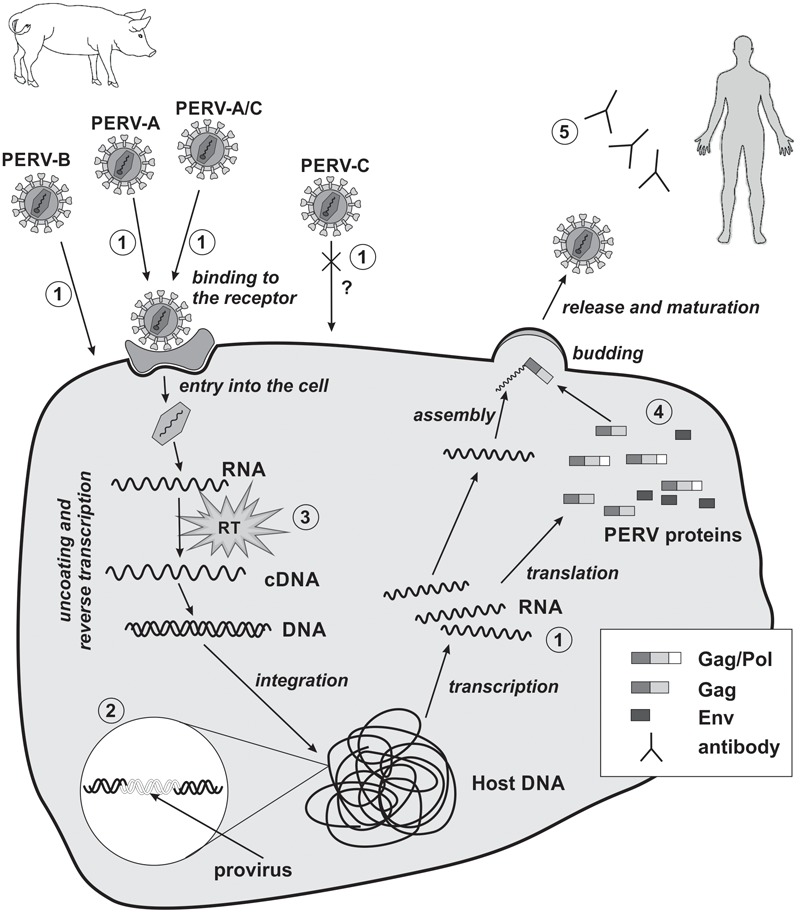
Replication cycle of PERV and strategies of PERV detection in xenotransplantation. 1, detection of viral RNA; 2, detection of viral DNA; 3, evaluation of reverse transcriptase activity; 4, detection of PERVs proteins; 5, detection of PERVs antibodies. RT, reverse transcriptase; cross and question mark, PERV-C there is no body of evidence for the possibility of human cells infection *in vivo*.

The replication cycle of PERVs is similar to that of other orthoretroviruses, especially gammaretroviruses such as MLV, and can be divided into early and late phases. The early phase includes adsorption onto the cell surface, entry into the cell, RT, and integration within the genome of the host cell (**Figure 2**). The late phase includes the expression of retrovirus genes, the release, and maturation of descendant virions.

### The Early Phase

The early phase begins with the binding of PERV SU to the appropriate receptor on the host cell. So far, only receptor for PERV-A has been identified. In pigs, it is porcine PERV-A receptor (PoPAR) (Ericsson et al., 2003). In humans, two PERV-A receptors have been identified: human PERV-A receptor 1 (HuPAR-1) and human PERV-A receptor 2 (HuPAR-2), also known as G-protein-coupled receptors 172A and 172B (Mazari et al., 2009, 2012; Nakaya et al., 2011) and as solute carrier family 52 members 2 and 1 (SLC52A2 and SLC52A1), respectively (Yonezawa and Inui, 2013). HuPAR-1 and HuPAR-2 are mammalian riboflavin transporters and are also known as human riboflavin transporter 3 (hRFT3) and human riboflavin transporter 1 (hRFT1), respectively (Yonezawa et al., 2008; Yao et al., 2010). These receptors are encoded by genes located on chromosomes 8 and 17, respectively, and are widespread in most of the human tissues (Ericsson et al., 2003), although the expression of *huPAR-1* is more ubiquitous (Yonezawa et al., 2008; Marcucci et al., 2009; Yao et al., 2010). The expression of *huPAR-2* is especially increased in the placenta and the small intestine. In contrast, the expression of *huPAR-1* is particularly enhanced in the brain and salivary glands (Yao et al., 2010; Nakaya et al., 2011). HuPAR-1 (445 amino acids) and HuPAR-2 (448 amino acids) are transmembrane proteins that share 86.7% sequence identity. HuPAR-2 is much more functional than HuPAR-1 in terms of PERV-A infection (Marcucci et al., 2009). There are two regions in HuPAR-2’s structure that are crucial for PERV-A infection. The first region lies within the first N-terminal 135 amino acids. It contains an absolute determinant of viral envelope binding – the leucine 109 (L109) as well as seven additional single residues that enhance the efficiency of PERV-A entry without any impact on envelope binding. The second region is located in the middle of HuPAR-2 (a.a. 152–285). This region is responsible for the 11-fold function compared to HuPAR-1 and has no effect on PERV envelope binding (Marcucci et al., 2009). The transcription of *huPAR-2* is controlled by DNA methylation and histone modification (Nakaya et al., 2011). Transcription factor activator protein-2γ (TFAP 2γ) is one of the transcription factors involved in the expression of *huPAR-2* in cytotrophoblast cells (Nakaya et al., 2012).

It was shown that PERV-A, -B, and -C can infect human and rodent cells that lack functional PERV receptors by using transactivation mechanism (Lavillette and Kabat, 2004). The alternative method of infection requires the SU glycoproteins of other gammaretroviruses containing a proline, histidine, glutamine (PHQ) motif or RBD domains and adequate receptors. The PHQ motif is located on the N-terminal region of SU in most gammaretroviruses and is important for virus infectiveness. Histidine is a very important component of this subunit. The mutation or deletion of this amino acid suppresses virus infectiveness and membrane fusion, preserving receptor-binding capability and the incorporation of the Env glycoprotein into the virions. PERVs contain only a portion of the PHQ motif, the tenth histidine (H10). The H10A (histidine↔alanine) mutation suppresses PERV infectivity. However, noninfectious PERVs can be transactivated by adding PHQ-containing SU glycoproteins or soluble RBDs from GaLV. One requirement for this transactivation is a functional GaLV receptor on the cells. Transactivation via GaLV RBD substantially enhances the infectivity of wild-type PERVs, even for cells with PERV receptors. Thus, limited tropism can be overcome with the use of the receptors and domains of other viruses (Lavillette and Kabat, 2004).

The binding of SU with its cell surface receptor induces conformational changes in the gp70 protein as well as its release from the fusion peptide of the TM subunit. As a consequence, the viral Env fuses with the host cell membrane, and the genetic material of the retrovirus covered by the CA enters the cell (Kubo et al., 2012). In the cytoplasm of the host cell, retroviral RNA is transcribed into dsDNA by retroviral RT within the reverse transcription complex (RTC). The RTC is comprised of genomic RNA, CA, p12 protein, NC, RT, IN, and cellular proteins. CA uncoating occurs after the entry into the host cell and is probably correlated with the RT process (Fassati, 2012). In the case of MLV uncoating happens after nuclear entry following mitosis. After dsDNA synthesis, the RTC changes into the pre-integration complex (PIC), which contains dsDNA as well as proteins from the virus (IN and CA) and the host. Gammaretroviral infection requires dividing cells, when the nuclear membrane has broken down. PIC enters the nucleus and subsequently dsDNA integrates within the host genome forming a stable proviral structure (Fassati, 2012; Rein, 2013). In the model proposed for MLV, PIC binds to chromosomes during mitosis with the help of p12. After mitosis, PIC is released from chromatin and viral DNA is built into the interphase chromatin with the aid of the IN (Rein, 2013). The integration of PERV into the human genome (cell line HEK293) is strongly enhanced at sites enriched in CpG islands, especially at transcriptional start sites. These integration sites are flanked by an eight-base palindromic consensus sequence, TG(int)GTACCAGC (Moalic et al., 2006).

### The Late Phase

Once integrated, the provirus DNA is transcribed by the host machinery to produce descendent retroviruses. The originated mRNA can be used for the production of viral proteins, or become the genomic RNA of new viral particles. The binding of Gag polyproteins to viral genomic RNA occurs in the cytoplasm and is accomplished through the interaction between the NC portion of Gag and a viral RNA ψ sequence. The ψ sequence of PERV has been characterized (Choi et al., 2015). Ensuing Gag multimers interact with the cellular membrane, initiating the budding process. Env (SU–TM) glycoproteins then begin to accumulate in the cell membrane (Martin-Serrano and Neil, 2011; Rein, 2011). During the docking and release of the nascent virion, L-domains play a very important role. L-Domains are short, conserved amino acid motifs in the p12 protein of the Gag polyprotein. The inactivation of this domain leads to the incomplete maturation of the released virions, the reduction of their release, and the accumulation of Gag polyprotein under the cellular membrane (Martin-Serrano and Neil, 2011). The L-domain exploits the cellular proteins to enhance the assembly and release of virions. Several proteins are involved in the budding of PERVs, including WW domain-containing protein 2 (WWP2), tumor susceptibility gene 101 (TSG101), and vacuolar protein sorting-associated protein 4 (VPS4) (Abe et al., 2014). After the release of the virions from the host cell, PR cleaves Gag polyprotein into minor subunits: MA, CA, and NC. PR also removes the C-terminal residues from the cytoplasmatic tail of the TM protein (R peptide), which is part of the Env protein. In this way, immature PERV particles are converted into mature, infectious virions (Bobkova et al., 2002).

## PERVS and Xenotransplantation Risk

The ability of PERVs to infect human cells *in vitro* raises concerns, especially in the context of the eventual use of porcine cells, tissues, and organs in xenotransplantation. The possibility of PERV transmission to various human cells, including peripheral blood mononuclear cells (PBMCs) (Clémenceau et al., 2001; Specke et al., 2001; Güell et al., 2017), embryonic kidney cell (HEK-293) line (Martin et al., 1998; Clémenceau et al., 2001; Specke et al., 2001; Lee et al., 2008a; Prabha and Verghese, 2012; Yue et al., 2015), and normal dermal human fibroblasts (NHDFs) (Kimsa et al., 2013) has been confirmed *in vitro*. However, PERV transmission *in vivo* has not been reported among patients with type 1 diabetes, after pancreatic islets xenografts (Heneine et al., 1998; Garkavenko et al., 2004; Valdes-Gonzalez et al., 2010; Matsumoto et al., 2016; Morozov et al., 2017), recipients of pig’s nerve cells (Fink et al., 2000), patients with porcine liver cell-based bioartificial liver (Di Nicuolo et al., 2010), porcine skin graft recipients (Scobie et al., 2013), and butchers exposed to contact with pig tissues (Garkavenko et al., 2004; Bittmann et al., 2012). It is possible, that in the case of *in vitro* studies, we are not able to reproduce the complicated dependence networks that have a significant impact on the defense of cells against PERV infection *in vivo*.

Currently, the risk of PERV transmission is considered to be low, assuming that the pigs are adequately and continuously monitored. To minimize the risk of PERV transmission during human xenotransplantation, donor pigs should be selected based on the absence of PERV-C and the lowest expression of PERV-A and -B. Biological materials such as animal saliva or blood should be used for screening. However, if the number of PERV copies in the organ for xenotransplantation differs compared to the material used for screening, an investigation of the whole animal or of its sisters or brothers should be performed (Denner, 2016b). Precise knowledge of the structure and replication cycle of PERVs is a prerequisite for planning strategies for PERV detection (Denner, 2011; Argaw and Wilson, 2012; Gola and Mazurek, 2014; Kimsa et al., 2014a; Godehardt et al., 2015). Such detection should be performed at the genomic, transcriptomic, and proteomic levels using methods with adequate sensitivity and specificity. Polymerase chain reaction (PCR), with the use of the primers complementary to the conserved regions of the *gag* and *pol* genes, permits the detection of PERV proviruses in the analyzed biological material. The virus subtype (PERV-A, -B, or -C) can be determined with the primers complementary to *env* gene. Concomitantly the potential risk of recombination between the subtypes can be assessed (PERV-A/C). Primers complementary to the LTRs can serve for the amplification of the whole genome of PERV provirus (Machnik et al., 2005; Sypniewski et al., 2005; Kim et al., 2009). RT-PCR, dependent on the analyzed biological material (cells or supernatant), permits the detection of RNA transcribed from the provirus genes or the presence of the viral genome (in particles). Determination of the RT activity further confirms the presence of the virus. Along with the increase of the number of copies of PERV RNA, RT activity can serve as a marker of the active replication cycle. Serology coupled with the use of western blot, ELISA, and immunofluorescence methods complements the diagnostics and can confirm PERV infection (**Figure 2**; Denner, 2011; Argaw and Wilson, 2012; Kimsa et al., 2014a). Visualization by transmission and scanning electron microscopy is also an effective method to confirm the presence of viral particles and their release from infected cells (Armstrong et al., 1971). The genetic material of the virus can also be detected with the use of the *in situ* hybridization technique (Wood et al., 2004). This method allows to determine the location of the viral nucleic acid and the percentage of infected cells, giving the opportunity to estimate the viral replication ability and the degree of integration in the host’s DNA.

The risk of viral infections due to the use of pigs as donors is estimated to be lower than in the case of human allografts. The use of human donors also carries the risk of transmission of viral infections, such as cytomegalovirus (CMV), viral hepatitis B (HBV), C (HCV), E (HEV), or Epstein–Barr virus (EBV) (Wynyard et al., 2014; Denner and Mueller, 2015; Denner, 2015, 2016b; Denner and Mankertz, 2017; Cooper et al., 2017; Meier et al., 2017). The urgency of allotransplantation may preclude complex diagnostics in the search for all possible human pathogens. It should also be noted that the immunological barriers of the recipient are substantially depleted by immunosuppression. In the case of xenotransplantation, pigs devoid of pathogens should be bred to ensure the safety of the procedure.

Even a small change in the genetic code of the PERV may be dangerous (Gemeniano et al., 2006; Argaw et al., 2008). Moreover, the barriers associated with the tropism of PERVs to human cells can be overcome with the use of other receptors or their corresponding domains from other viruses or by recombination with other PERV subtypes (Harrison et al., 2004; Lavillette and Kabat, 2004).

Eventual incorporation of the PERV within the human genome carries the risk that in the case of cell stimulation by other microorganisms, the expression of the virus might change leading to unknown consequences. Lipopolysacharide (LPS) is the most biologically active component of Gram-negative bacteria responsible for the pathophysiological effects associated with infection. *In vitro* studies revealed that LPS can promote virus production or can strongly inhibit virus integration in NHDF cell lines (Kimsa et al., 2013, 2014b).

The characteristic feature of retroviruses—the transcription of genomic RNA into the dsDNA and its incorporation within the host genome—represents a significant threat. It is well known that ERVs are present as a part of the vertebrate genome; however, it is unclear whether they have evolved from an exogenous ancestor or an LTR retrotransposon (Hayward, 2017). ERVs can influence the regulation of expression at genomic and proteomic levels (Galbraith et al., 2000; Stephan et al., 2001; Costa et al., 2014; Wynyard et al., 2014). Expression of the ERVs’ Env proteins, called syncytins, plays an important role in the development of the placenta. Envelope proteins provide fusogenic activity for the syncytiotrophoblast formation and regulate its homeostasis (Denner, 2016a). Their immunosuppressive properties contribute to preventing the rejection of the semiallotransplant embryo (Denner, 2016a). ERVs can influence the regulation of the innate immunity (Chuong et al., 2016), and some may even protect their hosts against viral infection (Malfavon-Borja and Feschotte, 2015). It has been estimated that about 8% of the human genome consists of retroviral sequences. Potential recombination of one of the three subtypes of PERVs with closely related human ERVs (HERVs), especially HERV-R or HERV-E, cannot be excluded, especially if both elements are located near to one another. Such a situation could theoretically give rise to a new virus with unknown pathogenic potential. Recombination could occur during packaging of PERV and HERV transcripts into a single retroviral particle. Changes in the expression of HERV-W genes have been observed in studies of the human embryonic kidney HEK 293 cell line exposed to PERVs. Both the mRNA and protein abundance of Env were significantly higher than in the control cells (Machnik et al., 2014). Conversely, studies on infected HEK 293 cells indicated that the recombination potential of PERVs and HERVs was low (Suling et al., 2003). Additionally, the evolution gap of about 20 million years between the time of PERV and HERV incorporation into the host genome probably permitted primates to develop adequate defense mechanisms for inactivating foreign ERVs, for example, restriction factors such as apolipoprotein B mRNA editing enzyme catalytic subunit 3 (APOBEC3) (Denner, 2016b). However, the final exclusion of PERV–HERV recombination would necessitate experiments *in vivo* (Suling et al., 2003).

The strategies of elimination the potential risks related with PERVs rely on the search of specific vaccines (Kaulitz et al., 2011), the use of antiretroviral drugs (mainly azidothymidine AZT) (Denner, 2017), attempts to reduce the PERVs expression by RNA interference (Semaan et al., 2012), or inactivation of all PERV proviruses in the pig genome by the CRISPR/Cas technique (Yang et al., 2015; Niu et al., 2017). With the application of the CRISPR-Cas9 technique, 62 copies of PERV’s gene *pol* were deactivated, leading to a 1000 times reduction in the virus ability to infect human cells (Yang et al., 2015). Long-term studies are underway to monitor the impact of PERV inactivation and gene editing on PERV-inactivated pigs (Niu et al., 2017). However, the question of whether such genetically engineered pigs could serve in the future as a safe resource of tissues and organs for xenotransplantation remains open (Denner, 2016b; Walters and Burlak, 2017).

## Concluding Remarks

In the near future, the targeted genetic modification of pigs may allow animals to be personalized for the medical needs of specific patients, optimizing treatments and lowering costs. In addition to all the typical safety measures preceding xenotransplantation, clinical trials should be followed by rigorous and lifelong monitoring of the recipient. Better understanding of the PERV replication strategy could contribute to elaborate medical procedures for use in xenotransplantation. Methods should be found preventing the entry of PERVs into cells and blocking the virus at every stage of its replication cycle. Much work is needed before xenotransplantation becomes a safe medical procedure.

## Author Contributions

The manuscript was equally drafted, revised, and approved by KŁ, EW, RN, PŁ, and UM.

## Conflict of Interest Statement

The authors declare that the research was conducted in the absence of any commercial or financial relationships that could be construed as a potential conflict of interest.

## References

[B1] Abe M. Fukuma A. Yoshikawa R. Miyazawa T. Yasuda J. (2014). Inhibition of budding/release of porcine endogenous retrovirus. *Microbiol. Immunol.* 58 432–438. 10.1111/1348-0421.12166 24931347

[B2] Akiyoshi D. E. Denaro M. Zhu H. Greenstein J. L. Banerjee P. Fishman J. A. (1998). Identification of a full-length cDNA for an endogenous retrovirus of miniature swine. *J. Virol.* 72 4503–4507. 9557749 10.1128/jvi.72.5.4503-4507.1998 PMC109691

[B3] Argaw T. Figueroa M. Salomon D. R. Wilson C. A. (2008). Identification of residues outside of the receptor binding domain that influence the infectivity and tropism of porcine endogenous retrovirus. *J. Virol.* 82 7483–7491. 10.1128/JVI.00295-08 18508891 PMC2493329

[B4] Argaw T. Wilson C. A. (2012). *Methods and Tools for Detection and Evaluation of the Risks of Porcine Endogenous Retrovirus in Porcine to Human Xenotransplantation.* Rijeka: InTech 10.5772/28799

[B5] Armstrong J. A. Porterfield J. S. De Madrid A. T. (1971). C-type virus particles in pig kidney cell lines. *J. Gen. Virol.* 10 195–198. 10.1099/0022-1317-10-2-195 4324256

[B6] Bittmann I. Mihica D. Plesker R. Denner J. (2012). Expression of porcine endogenous retroviruses (PERV) in different organs of a pig. *Virology* 433 329–336. 10.1016/j.virol.2012.08.030 22975674

[B7] Blusch J. H. Seelmeir S. von der Helm K. (2002). Molecular and enzymatic characterization of the porcine endogenous retrovirus protease. *J. Virol.* 76 7913–7917. 10.1128/JVI.76.15.7913-7917.2002 12097607 PMC136392

[B8] Bobkova M. Stitz J. Engelstädter M. Cichutek K. Buchholz C. J. (2002). Identification of R-peptides in envelope proteins of C-type retroviruses. *J. Gen. Virol.* 83 2241–2246. 10.1099/0022-1317-83-9-2241 12185279

[B9] Breese S. S. Jr. (1970). Virus-like particles occurring in cultures of stable pig kidney cell lines. *Arch. Gesamte Virusforsch.* 30 401–404. 10.1007/BF01258369 4195629

[B10] Chiang C.-Y. Pan Y.-R. Chou L.-F. Fang C.-Y. Wang S.-R. Yang C.-Y. (2007). Functional epitopes on porcine endogenous retrovirus envelope protein interacting with neutralizing antibody combining sites. *Virology* 361 364–371. 10.1016/j.virol.2006.11.016 17222436

[B11] Choi J. Kim H. Yoon J. K. Cho Y. Lee H.-J. Kim K. C. (2015). Identification of porcine endogenous retrovirus (PERV) packaging sequence and development of PERV packaging viral vector system. *J. Microbiol.* 53 348–353. 10.1007/s12275-015-5134-0 25935307

[B12] Chuong E. B. Elde N. C. Feschotte C. (2016). Regulatory evolution of innate immunity through co-option of endogenous retroviruses. *Science* 351 1083–1087. 10.1126/science.aad5497 26941318 PMC4887275

[B13] Clémenceau B. Jégou D. Martignat L. Saï P. (2001). Long-term follow-up failed to detect in vitro transmission of full-length porcine endogenous retroviruses from specific pathogen-free pig islets to human cells. *Diabetologia* 44 2044–2055. 10.1007/s001250100010 11719837

[B14] Cooper D. K. C. Gaston R. Eckhoff D. Ladowski J. Yamamoto T. Wang L. (2017). Xenotransplantation-the current status and prospects. *Br. Med. Bull.* 125 5–14. 10.1093/bmb/ldx043 29228112 PMC6487536

[B15] Costa M. R. Fischer N. Gulich B. Tönjes R. R. (2014). Comparison of porcine endogenous retroviruses infectious potential in supernatants of producer cells and in cocultures. *Xenotransplantation* 21 162–173. 10.1111/xen.12081 24447212

[B16] Czauderna F. Fischer N. Boller K. Kurth R. Tonjes R. R. (2000). Establishment and characterization of molecular clones of porcine endogenous retroviruses replicating on human cells. *J. Virol.* 74 4028–4038. 10.1128/JVI.74.9.4028-4038.2000 10756014 PMC111916

[B17] Dekker S. Toussaint W. Panayotou G. de Wit T. Visser P. Grosveld F. (2003). Intracellularly expressed single-domain antibody against p15 matrix protein prevents the production of porcine retroviruses. *J. Virol.* 77 12132–12139. 10.1128/JVI.77.22.12132-12139.2003 14581550 PMC254262

[B18] Denner J. (2008). Recombinant porcine endogenous retroviruses (PERV-A/C): a new risk for xenotransplantation? *Arch. Virol.* 153 1421–1426. 10.1007/s00705-008-0141-7 18584115

[B19] Denner J. (2011). Infectious risk in xenotransplantation - what post-transplant screening for the human recipient?: Infectious risk in xenotransplantation. *Xenotransplantation* 18 151–157. 10.1111/j.1399-3089.2011.00636.x 21696444

[B20] Denner J. (2015). Xenotransplantation and Hepatitis E virus. *Xenotransplantation* 22 167–173. 10.1111/xen.12156 25676629

[B21] Denner J. (2016a). Expression and function of endogenous retroviruses in the placenta. *APMIS* 124 31–43. 10.1111/apm.12474 26818260

[B22] Denner J. (2016b). How active are porcine endogenous retroviruses (PERVs)? *Viruses* 8:E215. 10.3390/v8080215 27527207 PMC4997577

[B23] Denner J. (2017). Can antiretroviral drugs be used to treat porcine endogenous retrovirus (PERV) infection after xenotransplantation? *Viruses* 9:E213. 10.3390/v9080213 28786944 PMC5580470

[B24] Denner J. Mankertz A. (2017). Porcine circoviruses and xenotransplantation. *Viruses* 9:E83. 10.3390/v9040083 28425928 PMC5408689

[B25] Denner J. Mueller N. J. (2015). Preventing transfer of infectious agents. *Int. J. Surg.* 23 306–311. 10.1016/j.ijsu.2015.08.032 26316157 PMC7185644

[B26] Denner J. Specke V. Thiesen U. Karlas A. Kurth R. (2003). Genetic alterations of the long terminal repeat of an ecotropic porcine endogenous retrovirus during passage in human cells. *Virology* 314 125–133. 10.1016/S0042-6822(03)00428-8 14517066

[B27] Denner J. Tonjes R. R. (2012). Infection barriers to successful xenotransplantation focusing on porcine endogenous retroviruses. *Clin. Microbiol. Rev.* 25 318–343. 10.1128/CMR.05011-11 22491774 PMC3346299

[B28] Dhanasekaran M. George J. J. Loganathan G. Narayanan S. Hughes M. G. Williams S. K. (2017). Pig islet xenotransplantation. *Curr. Opin. Organ. Transplant.* 22 452–462. 10.1097/MOT.0000000000000455 28759462

[B29] Di Nicuolo G. D’Alessandro A. Andria B. Scuderi V. Scognamiglio M. Tammaro A. (2010). Long-term absence of porcine endogenous retrovirus infection in chronically immunosuppressed patients after treatment with the porcine cell-based academic medical center bioartificial liver: absence of PERV after BAL treatment. *Xenotransplantation* 17 431–439. 10.1111/j.1399-3089.2010.00617.x 21158944

[B30] Ekser B. Cooper D. K. (2010). Overcoming the barriers to xenotransplantation: prospects for the future. *Expert Rev. Clin. Immunol.* 6 219–230. Available at: https://www.ncbi.nlm.nih.gov/pmc/articles/PMC2857338/ [Accessed March 2, 2018]. 20402385 10.1586/eci.09.81 PMC2857338

[B31] Ericsson T. A. Takeuchi Y. Templin C. Quinn G. Farhadian S. F. Wood J. C. (2003). Identification of receptors for pig endogenous retrovirus. *Proc. Natl. Acad. Sci. U.S.A.* 100 6759–6764. 10.1073/pnas.1138025100 12740431 PMC164520

[B32] Fassati A. (2012). Multiple roles of the capsid protein in the early steps of HIV-1 infection. *Virus Res.* 170 15–24. 10.1016/j.virusres.2012.09.012 23041358

[B33] Fink J. S. Schumacher J. M. Ellias S. L. Palmer E. P. Saint-Hilaire M. Shannon K. (2000). Porcine xenografts in Parkinson’s disease and Huntington’s disease patients: preliminary results. *Cell Transplant.* 9 273–278. 10.1177/096368970000900212 10811399

[B34] Galbraith D. N. Kelly H. T. Dyke A. Reid G. Haworth C. Beekman J. (2000). Design and validation of immunological tests for the detection of Porcine endogenous retrovirus in biological materials. *J. Virol. Methods* 90 115–124. 10.1016/S0166-0934(00)00200-7 11064112

[B35] Garkavenko O. Croxson M. C. Irgang M. Karlas A. Denner J. Elliott R. B. (2004). Monitoring for presence of potentially xenotic viruses in recipients of pig islet xenotransplantation. *J. Clin. Microbiol.* 42 5353–5356. 10.1128/JCM.42.11.5353-5356.2004 15528741 PMC525280

[B36] Gemeniano M. Mpanju O. Salomon D. R. Eiden M. V. Wilson C. A. (2006). The infectivity and host range of the ecotropic porcine endogenous retrovirus, PERV-C, is modulated by residues in the C-terminal region of its surface envelope protein. *Virology* 346 108–117. 10.1016/j.virol.2005.10.021 16309725

[B37] Godehardt A. W. Rodrigues Costa M. Tönjes R. R. (2015). Review on porcine endogenous retrovirus detection assays–impact on quality and safety of xenotransplants. *Xenotransplantation* 22 95–101. 10.1111/xen.12154 25641488 PMC4413356

[B38] Gola J. Mazurek U. (2014). Detection of porcine endogenous retrovirus in xenotransplantation. *Reprod. Biol.* 14 68–73. 10.1016/j.repbio.2014.01.006 24607257

[B39] Güell M. Niu D. Kan Y. George H. Wang T. Lee I.-H. (2017). PERV inactivation is necessary to guarantee absence of pig-to-patient PERVs transmission in xenotransplantation. *Xenotransplantation* 24:e12366. 10.1111/xen.12366 29171094

[B40] Ha H.-S. Huh J.-W. Kim D.-S. Kang D.-W. Cho B.-W. Kim H.-S. (2007). Promoter activity of the long terminal repeats of porcine endogenous retroviruses of the Korean domestic pig. *Mol. Cells* 24 148–151. 17846510

[B41] Ha H.-S. Lee Y.-C. Park S.-J. Jung Y.-D. Ahn K. Moon J.-W. (2012). In vitro CpG methylation and garcinol reduce PERV LTR promoter activity. *Genes Genomics* 34 217–222. 10.1007/s13258-011-0161-7

[B42] Hara H. Cooper D. K. C. (2011). Xenotransplantation – the future of corneal transplantation? *Cornea* 30 371–378. 10.1097/ICO.0b013e3181f237ef 21099407 PMC3081421

[B43] Harrison I. Takeuchi Y. Bartosch B. Stoye J. P. (2004). Determinants of high titer in recombinant porcine endogenous retroviruses. *J. Virol.* 78 13871–13879. 10.1128/JVI.78.24.13871-13879.2004 15564495 PMC533952

[B44] Hayward A. (2017). Origin of the retroviruses: when, where, and how? *Curr. Opin. Virol.* 25 23–27. 10.1016/j.coviro.2017.06.006 28672160 PMC5962544

[B45] Heneine W. Tibell A. Switzer W. M. Sandstrom P. Rosales G. V. Mathews A. (1998). No evidence of infection with porcine endogenous retrovirus in recipients of porcine islet-cell xenografts. *Lancet* 352 695–699. 10.1016/S0140-6736(98)07145-1 9728986

[B46] Hryhorowicz M. Zeyland J. Słomski R. Lipiński D. (2017). Genetically modified pigs as organ donors for xenotransplantation. *Mol. Biotechnol.* 59 435–444. 10.1007/s12033-017-0024-9 28698981 PMC5617878

[B47] Huh J.-W. Cho B.-W. Kim D.-S. Ha H.-S. Noh Y.-N. Yi J.-M. (2007). Long terminal repeats of porcine endogenous retroviruses in *Sus scrofa*. *Arch. Virol.* 152 2271–2276. 10.1007/s00705-007-1049-3 17823769

[B48] Jung K. C. Simond D. M. Moran C. Hawthorne W. J. Jeon J. T. Jin D. I. (2008). Investigation of deletion variation and methylation patterns in the 5’ LTR of porcine endogenous retroviruses. *Asian Aust. J. Anim. Sci.* 21 1572–1575. 10.5713/ajas.2008.80065

[B49] Jung Y.-D. Ha H.-S. Park S.-J. Oh K.-B. Im G.-S. Kim T.-H. (2013a). Identification and promoter analysis of PERV LTR subtypes in NIH-miniature pig. *Mol. Cells* 35 99–105. 10.1007/s10059-013-2289-6 23456331 PMC3887905

[B50] Jung Y.-D. Lee J.-R. Kim Y.-J. Ha H.-S. Oh K.-B. Im G.-S. (2013b). Promoter activity analysis and methylation characterization of LTR elements of PERVs in NIH miniature pig. *Genes Genet. Syst.* 88 135–142. 23832305 10.1266/ggs.88.135

[B51] Karlas A. Irgang M. Votteler J. Specke V. Ozel M. Kurth R. (2010). Characterisation of a human cell-adapted porcine endogenous retrovirus PERV-A/C. *Ann. Transplant.* 15 45–54. 20657519

[B52] Kaulitz D. Fiebig U. Eschricht M. Wurzbacher C. Kurth R. Denner J. (2011). Generation of neutralising antibodies against porcine endogenous retroviruses (PERVs). *Virology* 411 78–86. 10.1016/j.virol.2010.12.032 21237477

[B53] Kim J. H. Choi E. Y. Jung E.-S. Kwon Y. Lee D. S. Hwang D. Y. (2009). Characterization of clones of human cell line infected with porcine endogenous retrovirus (PERV) from porcine cell line, PK-15. *Infect. Chemother.* 41 1–8. 10.3947/ic.2009.41.1.1

[B54] Kim M. K. Hara H. (2015). Current status of corneal xenotransplantation. *Int. J. Surg.* 23 255–260. 10.1016/j.ijsu.2015.07.685 26231995

[B55] Kimsa M. Strzalka-Mrozik B. Kimsa M. Gola J. Nicholson P. Lopata K. (2014a). Porcine endogenous retroviruses in xenotransplantation—molecular aspects. *Viruses* 6 2062–2083. 10.3390/v6052062 24828841 PMC4036542

[B56] Kimsa M. W. Strzalka-Mrozik B. Kimsa M. C. Mazurek U. Kruszniewska-Rajs C. Gola J. (2014b). Differential expression of tripartite motif-containing family in normal human dermal fibroblasts in response to porcine endogenous retrovirus infection. *Folia Biol.* 60 144–151. 25056437 10.14712/fb2014060030144

[B57] Kimsa M. C. Strzałka-Mrozik B. Kimsa M. W. Kruszniewska-Rajs C. Gola J. Adamska J. (2013). Porcine endogenous retrovirus infection changes the expression of inflammation-related genes in lipopolysaccharide-stimulated human dermal fibroblasts. *Ann. Transplant.* 18 576–586. 10.12659/AOT.889310 24157628

[B58] Krach U. Fischer N. Czauderna F. Tonjes R. R. (2001). Comparison of replication-competent molecular clones of porcine endogenous retrovirus class A and class B derived from pig and human cells. *J. Virol.* 75 5465–5472. 10.1128/JVI.75.12.5465-5472.2001 11356953 PMC114258

[B59] Kubo Y. Hayashi H. Matsuyama T. Sato H. Yamamoto N. (2012). Retrovirus entry by endocytosis and cathepsin proteases. *Adv. Virol.* 2012 1–14. 10.1155/2012/640894 23304142 PMC3523128

[B60] Lavillette D. Kabat D. (2004). Porcine endogenous retroviruses infect cells lacking cognate receptors by an alternative pathway: implications for retrovirus evolution and xenotransplantation. *J. Virol.* 78 8868–8877. 10.1128/JVI.78.16.8868-8877.2004 15280495 PMC479092

[B61] Lee D. Kim N. Y. Bae G.-E. Lee H. J. Kwon M. Kim S. S. (2008a). Transmissible infection of human 293T cells with porcine endogenous retroviruses subgroup a from NIH-miniature pig. *Transplant. Proc.* 40 3742–3745. 10.1016/j.transproceed.2008.09.035 19100479

[B62] Lee D. Lee J. Park N. Oh Y.-K. Kwon M. Kim Y. B. (2008b). Analysis of natural recombination in porcine endogenous retrovirus envelope genes. *J. Microbiol. Biotechnol.* 18 585–590. 18388481

[B63] Lee D. Lee J. Uhm S. J. Lee Y. S. Park M. J. Park H. Y. (2006). Molecular characterization of the porcine endogenous retrovirus subclass A and B envelope gene from pigs. *Transplant. Proc.* 38 3066–3069. 10.1016/j.transproceed.2006.08.144 17112901

[B64] Lee Y. J. Park S.-H. Bae E. H. Jung Y.-T. (2012). Characterization of molecular clones of porcine endogenous retrovirus-A containing different numbers of U3 repeat boxes in the long terminal repeat region. *J. Virol. Methods* 181 103–108. 10.1016/j.jviromet.2012.01.023 22343070

[B65] Liu G. Li Z. Pan M. Ge M. Wang Y. Gao Y. (2011). Genetic prevalence of porcine endogenous retrovirus in chinese experimental miniature pigs. *Transplant. Proc.* 43 2762–2769. 10.1016/j.transproceed.2011.06.061 21911159

[B66] Luo Y. Lin L. Bolund L. Jensen T. G. Sørensen C. B. (2012). Genetically modified pigs for biomedical research. *J. Inherit. Metab. Dis.* 35 695–713. 10.1007/s10545-012-9475-0 22453682

[B67] Ma Y. Lv M. Xu S. Wu J. Tian K. Zhang J. (2010a). Identification of full-length proviral DNA of porcine endogenous retrovirus from Chinese Wuzhishan miniature pigs inbred. *Comp. Immunol. Microbiol. Infect. Dis.* 33 323–331. 10.1016/j.cimid.2008.10.007 19070900

[B68] Ma Y. Yang Y. Lv M. Yan Q. Zheng L. Ding F. (2010b). Real-time quantitative polymerase chain reaction with SYBR green I detection for estimating copy numbers of porcine endogenous retrovirus from Chinese miniature pigs. *Transplant. Proc.* 42 1949–1952. 10.1016/j.transproceed.2010.01.054 20620553

[B69] Machnik G. Klimacka-Nawrot E. Sypniewski D. Matczyńska D. Gałka S. Bednarek I. (2014). Porcine endogenous retrovirus (PERV) infection of HEK-293 cell line alters expression of human endogenous retrovirus (HERV-W) sequences. *Folia Biol.* 60 35–46. 24594055 10.14712/fb2014060010035

[B70] Machnik G. Sypniewski D. Wydmuch Z. Cholewa K. Mazurek U. Wilczok T. (2005). Sequence analysis of proviral DNA of porcine endogenous retroviruses. *Transplant. Proc.* 37 4610–4614. 10.1016/j.transproceed.2005.10.115 16387182

[B71] Magre S. Takeuchi Y. Bartosch B. (2003). Xenotransplantation and pig endogenous retroviruses. *Rev. Med. Virol.* 13 311–329. 10.1002/rmv.404 12931341

[B72] Malfavon-Borja R. Feschotte C. (2015). Fighting fire with fire: endogenous retrovirus envelopes as restriction factors. *J. Virol.* 89 4047–4050. 10.1128/JVI.03653-14 25653437 PMC4442362

[B73] Marcucci K. T. Argaw T. Wilson C. A. Salomon D. R. (2009). Identification of two distinct structural regions in a human porcine endogenous retrovirus receptor, HuPAR2 contributing to function for viral entry. *Retrovirology* 6:3. 10.1186/1742-4690-6-3 19144196 PMC2630988

[B74] Marcucci K. T. Martina Y. Harrison F. Wilson C. A. Salomon D. R. (2008). Functional hierarchy of two L domains in porcine endogenous retrovirus (PERV) that influence release and infectivity. *Virology* 375 637–645. 10.1016/j.virol.2008.02.017 18355887 PMC2626135

[B75] Martin U. Kiessig V. Blusch J. H. Haverich A. von der Helm K. Herden T. (1998). Expression of pig endogenous retrovirus by primary porcine endothelial cells and infection of human cells. *Lancet* 352 692–694. 10.1016/S0140-6736(98)07144-X 9728985

[B76] Martin-Serrano J. Neil S. J. D. (2011). Host factors involved in retroviral budding and release. *Nat. Rev. Microbiol.* 9 519–531. 10.1038/nrmicro2596 21677686

[B77] Matousková M. Vesely P. Daniel P. Mattiuzzo G. Hector R. D. Scobie L. (2013). Role of DNA methylation in expression and transmission of porcine endogenous retroviruses. *J. Virol.* 87 12110–12120. 10.1128/JVI.03262-12 23986605 PMC3807924

[B78] Matsumoto S. Tomiya M. Sawamoto O. (2016). Current status and future of clinical islet xenotransplantation. *J. Diabetes* 8 483–493. 10.1111/1753-0407.12395 26987992

[B79] Mazari P. M. Argaw T. Valdivieso L. Zhang X. Marcucci K. T. Salomon D. R. (2012). Comparison of the convergent receptor utilization of a retargeted feline leukemia virus envelope with a naturally-occurring porcine endogenous retrovirus A. *Virology* 427 118–126. 10.1016/j.virol.2012.02.012 22405627 PMC3572736

[B80] Mazari P. M. Linder-Basso D. Sarangi A. Chang Y. Roth M. J. (2009). Single-round selection yields a unique retroviral envelope utilizing GPR172A as its host receptor. *Proc. Natl. Acad. Sci. U.S.A.* 106 5848–5853. 10.1073/pnas.0809741106 19307586 PMC2667028

[B81] Mazurek U. Kimsa M. C. Strzalka-Mrozik B. Kimsa M. W. Adamska J. Lipinski D. (2013). Quantitative analysis of porcine endogenous retroviruses in different organs of transgenic pigs generated for xenotransplantation. *Curr. Microbiol.* 67 505–514. 10.1007/s00284-013-0397-3 23728786

[B82] Meier R. P. H. Muller Y. D. Balaphas A. Morel P. Pascual M. Seebach J. D. (2017). Xenotransplantation: back to the future? *Transpl. Int.* 10.1111/tri.13104 [Epub ahead of print]. 29210109

[B83] Moalic Y. Blanchard Y. Felix H. Jestin A. (2006). Porcine endogenous retrovirus integration sites in the human genome: features in common with those of murine leukemia virus. *J. Virol.* 80 10980–10988. 10.1128/JVI.00904-06 16928752 PMC1642138

[B84] Morozov V. A. Wynyard S. Matsumoto S. Abalovich A. Denner J. Elliott R. (2017). No PERV transmission during a clinical trial of pig islet cell transplantation. *Virus Res.* 227 34–40. 10.1016/j.virusres.2016.08.012 27677465

[B85] Nakaya Y. Shimode S. Kobayashi T. Imakawa K. Miyazawa T. (2012). Binding of transcription factor activating protein 2 γ on the 5′-proximal promoter region of human porcine endogenous retrovirus subgroup A receptor 2/GPR172B: binding of TFAP2γ on the HuPAR-2 proximal promoter. *Xenotransplantation* 19 177–185. 10.1111/j.1399-3089.2012.00701.x 22702469

[B86] Nakaya Y. Shojima T. Yasuda J. Imakawa K. Miyazawa T. (2011). Epigenetic regulation on the 5′-proximal CpG island of human porcine endogenous retrovirus subgroup A receptor 2/GPR172B. *Microbes Infect.* 13 49–57. 10.1016/j.micinf.2010.09.014 20951222

[B87] Nessler M. Chrapusta A. (2013). Możliwości zastosowania ksenogenicznych substytutów skóry w leczeniu oparzeń-przegląd piśmiennictwa. *Leczenie Ran* 10 47–52. 10.15374/lr2013008

[B88] Niebert M. Tonjes R. R. (2005). Evolutionary spread and recombination of porcine endogenous retroviruses in the suiformes. *J. Virol.* 79 649–654. 10.1128/JVI.79.1.649-654.2005 15596862 PMC538718

[B89] Niu D. Wei H.-J. Lin L. George H. Wang T. Lee I.-H. (2017). Inactivation of porcine endogenous retrovirus in pigs using CRISPR-Cas9. *Science* 357 1303–1307. 10.1126/science.aan4187 28798043 PMC5813284

[B90] Park S.-J. Huh J.-W. Kim D.-S. Ha H.-S. Jung Y.-D. Ahn K. (2010). Analysis of the molecular and regulatory properties of active porcine endogenous retrovirus gamma-1 long terminal repeats in kidney tissues of the NIH-Miniature pig. *Mol. Cells* 30 319–325. 10.1007/s10059-010-0121-0 20811814

[B91] Prabha S. M. Verghese S. (2012). Transmission of zoonoses in xenotransplantation: porcine endogenous retroviruses from an immunological and molecular point of view. *Indian J. Med. Sci.* 66 199–206. 10.4103/0019-5359.115210 23897566

[B92] Preuss T. Fischer N. Boller K. Tönjes R. R. (2006). Isolation and characterization of an infectious replication-competent molecular clone of ecotropic porcine endogenous retrovirus class C. *J. Virol.* 80 10258–10261. 10.1128/JVI.01140-06 17005704 PMC1617276

[B93] Reardon S. (2015). New life for pig-to-human transplants. *Nat. News* 527 152–154. 10.1038/527152a 26560282

[B94] Rein A. (2011). Murine leukemia viruses: objects and organisms. *Adv. Virol.* 2011:403419. 10.1155/2011/403419 22312342 PMC3265304

[B95] Rein A. (2013). Murine leukemia virus p12 functions include hitchhiking into the nucleus. *Proc. Natl. Acad. Sci. U.S.A.* 110 9195–9196. 10.1073/pnas.1307399110 23708120 PMC3677489

[B96] Scheef G. Fischer N. Flory E. Schmitt I. Tonjes R. R. (2002). Transcriptional regulation of porcine endogenous retroviruses released from porcine and infected human cells by heterotrimeric protein complex NF-Y and impact of immunosuppressive drugs. *J. Virol.* 76 12553–12563. 10.1128/JVI.76.24.12553-12563.2002 12438581 PMC136706

[B97] Scheef G. Fischer N. Krach U. Tonjes R. R. (2001). The number of a U3 repeat box acting as an enhancer in long terminal repeats of polytropic replication-competent porcine endogenous retroviruses dynamically fluctuates during serial virus passages in human cells. *J. Virol.* 75 6933–6940. 10.1128/JVI.75.15.6933-6940.2001 11435573 PMC114421

[B98] Scobie L. Padler-Karavani V. Le Bas-Bernardet S. Crossan C. Blaha J. Matouskova M. (2013). Long-term IgG response to porcine Neu5Gc antigens without transmission of PERV in burn patients treated with porcine skin xenografts. *J. Immunol.* 1950 2907–2915. 10.4049/jimmunol.1301195 23945141 PMC3782708

[B99] Semaan M. Kaulitz D. Petersen B. Niemann H. Denner J. (2012). Long-term effects of PERV-specific RNA interference in transgenic pigs. *Xenotransplantation* 19 112–121. 10.1111/j.1399-3089.2012.00683.x 22497513

[B100] Specke V. Rubant S. Denner J. (2001). Productive infection of human primary cells and cell lines with porcine endogenous retroviruses. *Virology* 285 177–180. 10.1006/viro.2001.0934 11437652

[B101] Stephan O. Schwendemann J. Specke V. Tacke S. J. Boller K. Denner J. (2001). Porcine endogenous retroviruses (PERVs): generation of specific antibodies, development of an immunoperoxidase assay (IPA) and inhibition by AZT. *Xenotransplantation* 8 310–316. 10.1034/j.1399-3089.2001.00098.x 11737857

[B102] Suling K. Quinn G. Wood J. Patience C. (2003). Packaging of human endogenous retrovirus sequences is undetectable in porcine endogenous retrovirus particles produced from human cells. *Virology* 312 330–336. 10.1016/S0042-6822(03)00380-5 12919738

[B103] Sypniewski D. Machnik G. Mazurek U. Wilczok T. Smorag Z. Jura J. (2005). Distribution of porcine endogenous retroviruses (PERVs) DNA in organs of a domestic pig. *Med. Sci. Monit. Basic Res.* 10 46–51. 16218033

[B104] Takeuchi Y. Patience C. Magre S. Weiss R. A. Banerjee P. T. Le Tissier P. (1998). Host range and interference studies of three classes of pig endogenous retrovirus. *J. Virol.* 72 9986–9991. 9811736 10.1128/jvi.72.12.9986-9991.1998 PMC110514

[B105] Tang H.-B. Ouyang K. Rao G.-B. Ma L. Zhong H. Bai A. (2016). Characterization of complete genome sequences of a porcine endogenous retrovirus isolated from China Bama Minipig reveals an evolutionary time earlier than that of isolates from European Minipigs. *Transplant. Proc.* 48 222–228. 10.1016/j.transproceed.2015.12.005 26915872

[B106] Tonjes R. R. Niebert M. (2003). Relative age of proviral porcine endogenous retrovirus sequences in *Sus scrofa* based on the molecular clock hypothesis. *J. Virol.* 77 12363–12368. 10.1128/JVI.77.22.12363-12368.2003 14581574 PMC254287

[B107] Valdes-Gonzalez R. Dorantes L. M. Bracho-Blanchet E. Rodríguez-Ventura A. White D. J. G. (2010). No evidence of porcine endogenous retrovirus in patients with type 1 diabetes after long-term porcine islet xenotransplantation. *J. Med. Virol.* 82 331–334. 10.1002/jmv.21655 20029803

[B108] Walters E. M. Burlak C. (2017). Xenotransplantation literature update, September/October 2017. *Xenotransplantation* 24:e12338. 10.1111/xen.12367 29168238

[B109] Watanabe R. Miyazawa T. Matsuura Y. (2005). Cell-binding properties of the envelope proteins of porcine endogenous retroviruses. *Microbes Infect.* 7 658–665. 10.1016/j.micinf.2005.01.008 15876545

[B110] Weiss R. A. (2006). The discovery of endogenous retroviruses. *Retrovirology* 3:67. 10.1186/1742-4690-3-67 17018135 PMC1617120

[B111] Wilson C. A. Laeeq S. Ritzhaupt A. Colon-Moran W. Yoshimura F. K. (2003). Sequence analysis of porcine endogenous retrovirus long terminal repeats and identification of transcriptional regulatory regions. *J. Virol.* 77 142–149. 10.1128/JVI.77.1.142-149.2003 12477819 PMC140639

[B112] Wolf G. Nielsen A. L. Mikkelsen J. G. Pedersen F. S. (2013). Epigenetic marking and repression of porcine endogenous retroviruses. *J. Gen. Virol.* 94 960–970. 10.1099/vir.0.049288-0 23324470

[B113] Wood J. C. Quinn G. Suling K. M. Oldmixon B. A. Van Tine B. A. Cina R. (2004). Identification of exogenous forms of human-tropic porcine endogenous retrovirus in miniature Swine. *J. Virol.* 78 2494–2501. 10.1128/JVI.78.5.2494-2501.2004 14963150 PMC369241

[B114] Wynyard S. Nathu D. Garkavenko O. Denner J. Elliott R. (2014). Microbiological safety of the first clinical pig islet xenotransplantation trial in New Zealand. *Xenotransplantation* 21 309–323. 10.1111/xen.12102 24801820

[B115] Yang L. Güell M. Niu D. George H. Lesha E. Grishin D. (2015). Genome-wide inactivation of porcine endogenous retroviruses (PERVs). *Science* 350 1101–1104. 10.1126/science.aad1191 26456528

[B116] Yao Y. Yonezawa A. Yoshimatsu H. Masuda S. Katsura T. Inui K.-I. (2010). Identification and comparative functional characterization of a new human riboflavin transporter hRFT3 expressed in the brain. *J. Nutr.* 140 1220–1226. 10.3945/jn.110.122911 20463145

[B117] Yonezawa A. Inui K. (2013). Novel riboflavin transporter family RFVT/SLC52: identification, nomenclature, functional characterization and genetic diseases of RFVT/SLC52. *Mol. Aspects Med.* 34 693–701. 10.1016/j.mam.2012.07.014 23506902

[B118] Yonezawa A. Masuda S. Katsura T. Inui K. (2008). Identification and functional characterization of a novel human and rat riboflavin transporter, RFT1. *Am. J. Physiol. Cell Physiol.* 295 C632–C641. 10.1152/ajpcell.00019.2008 18632736

[B119] Yu P. Zhang L. Li S. F. Cheng J. Q. Lu Y. R. Zeng Y. Z. (2007). A rapid method for detection of the copy number of porcine endogenous retrovirus in swine. *J. Rapid Methods Autom. Microbiol.* 15 199–205. 10.1007/s11626-009-9264-8 20108128

[B120] Yue S. Zhang Y. Gao Y. (2015). A study on the susceptibility of allogeneic human hepatocytes to porcine endogenous retrovirus. *Eur. Rev. Med. Pharmacol. Sci.* 19 3486–3491. 26439047

[B121] Zeyland J. Woźniak A. Gawrońska B. Juzwa W. Jura J. Nowak A. (2014). Double transgenic pigs with combined expression of human α1,2-fucosyltransferase and α-galactosidase designed to avoid hyperacute xenograft rejection. *Arch. Immunol. Ther. Exp.* 62 411–422. 10.1007/s00005-014-0280-3 24554032 PMC4164832

[B122] Zhang P. Yu P. Wang W. Zhang L. Li S. Bu H. (2010a). An effective method for the quantitative detection of porcine endogenous retrovirus in pig tissues. *Vitro Cell. Dev. Biol. Anim.* 46 408–410. 10.1007/s11626-009-9264-8 20108128

[B123] Zhang P. Yu P. Wang W. Zhang L. Li S. F. Bu H. (2010b). Molecular characterization of long terminal repeat of porcine endogenous retroviruses in Chinese pigs. *Acta Virol.* 54 165–172. 10.4149/av_2010_03_165 20822308

